# Cell-Penetrating Peptide-Mediated Biomolecule Transportation in Artificial Lipid Vesicles and Living Cells

**DOI:** 10.3390/molecules29143339

**Published:** 2024-07-16

**Authors:** Akari Miwa, Koki Kamiya

**Affiliations:** Division of Molecular Science, Graduate School of Science and Technology, Gunma University, Kiryu 376-8515, Gunma, Japan; t242a008@gunma-u.ac.jp

**Keywords:** membrane-active peptide, cell-penetrating peptide, lipid vesicle, artificial cell model, biomolecule transport, protein transport, therapy

## Abstract

Signal transduction and homeostasis are regulated by complex protein interactions in the intracellular environment. Therefore, the transportation of impermeable macromolecules (nucleic acids, proteins, and drugs) that control protein interactions is essential for modulating cell functions and therapeutic applications. However, macromolecule transportation across the cell membrane is not easy because the cell membrane separates the intra/extracellular environments, and the types of molecular transportation are regulated by membrane proteins. Cell-penetrating peptides (CPPs) are expected to be carriers for molecular transport. CPPs can transport macromolecules into cells through endocytosis and direct translocation. The transport mechanism remains largely unclear owing to several possibilities. In this review, we describe the methods for investigating CPP conformation, translocation, and cargo transportation using artificial membranes. We also investigated biomolecular transport across living cell membranes via CPPs. Subsequently, we show not only the biochemical applications but also the synthetic biological applications of CPPs. Finally, recent progress in biomolecule and nanoparticle transportation via CPPs into specific tissues is described from the viewpoint of drug delivery. This review provides the opportunity to discuss the mechanism of biomolecule transportation through these two platforms.

## 1. Introduction

**Figure 1:**
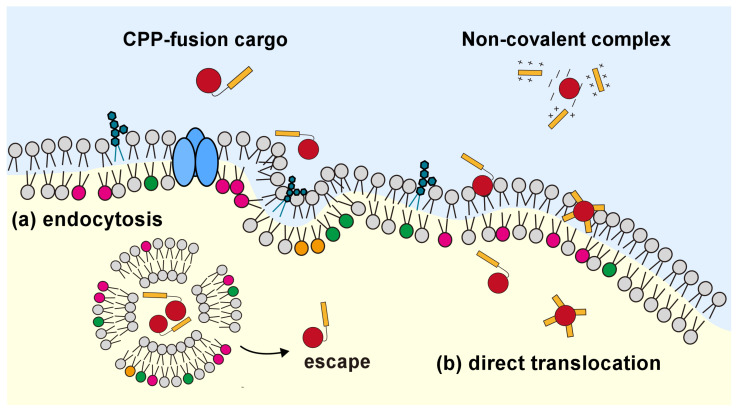
Schematic representation of CPP-mediated cargo molecule transportation into the living cell. Pink, orange and green heads show some lipids in the inner leaflet; PS, PI and PE, respectively.

The cell membrane separates the intracellular and extracellular environments. It comprises a phospholipid bilayer and integrates membrane proteins, such as ion channels [1,2], receptors [3,4], and transporters [5,6]. These proteins in the cell membrane regulate the transport of molecules entering and exiting the living cell to allow the transport of essential materials into the cell and discard waste products from the cell. This is called selective semi-permeability [7]. The intracellular environment contains various types of proteins, such as kinases [8,9], phosphatases [10,11], ligases [12,13], and transferases [14,15]. Smooth signal transduction [16] and homeostasis [17] are typically maintained because of complex interactions between proteins. Therefore, controlling these protein interactions outside of living cells is essential for investigating living cell systems and therapies. Several methods have been proposed for the translocation of biomolecules [18,19]. For example, to achieve low toxicity and efficient delivery, nanoparticles formed from cube-octameric silsecquioxanes (COSSs) and hydrogels formed from hyaluronic acid (HA) have been proposed for peptide or protein delivery [20,21,22]. Electroporation [23,24] and lipofection [25,26] are methods used for translocating nucleic acids into living cells. However, the problem with electroporation is that it induces cell lysis and death. Liposome-mediated translocations (such as lipofection) are entrapped in endosomes. To overcome these problems, the cell-penetrating peptide (CPP)-mediated translocation of biomolecules has been investigated (Figure 1). TAT was initially discovered as a CPP derived from the HIV-1 transactivator of the HIV-1 protein Tat [27,28]. When the fragment of Tat protein was conjugated to β-galactosidase and HRP, these enzymes were translocated into mouse model cells and HeLa cells. Cell-penetrating peptide-mediated biomolecule translocation has relatively low cytotoxicity compared with other translocation methods. Therefore, this peptide-mediated translocation system may have therapeutic applications [29,30,31].

Although translocation via CPPs has been widely reported, the translocation mechanism of large cargo molecules is not entirely understood; how CPPs enable the transport of biomolecules beyond their size is still unclear. The mechanism of CPP translocation and cargo transportation has been investigated using an artificial membrane consisting of only phospholipids, which easily changes the membrane composition [32,33] because cell membranes containing various types of lipids and membrane proteins are complex.

This review focuses on the methodologies for investigating CPP conformation, CPP translocation, and cargo transportation using two platforms: artificial membranes (lipid vesicles and planar lipid membranes) and living cells. In particular, we introduce biomolecular transport across artificial and living cell membranes. Therefore, this review provides the opportunity to discuss the mechanisms of biomolecular transportation. In addition, we introduce not only biochemical applications but also synthetic biological applications of CPPs. The combination of synthetic biology and cell-penetrating peptides will contribute to the construction of artificial cells and the elucidation of cargo transportation mechanisms. Finally, from the viewpoint of drug delivery, recent progress in biomolecule and nanoparticle transportation via CPPs that have tissue specificity is described.

## 2. Membrane-Active Peptides

Recently, short peptides of 5–50 amino acids were shown to have unique activity in terms of cell membrane permeability and attack on multidrug-resistant bacteria. In this section, we introduce the classification of membrane-active peptides, their mechanisms of translocation, and their antimicrobial properties.

### 2.1. Classification of Membrane-Active Peptides

Membrane-active peptides (MAPs) interact with membranes. The MAP classifications based on primary structures are listed in Table 1. Based on their apparent activities, there were two major classes. Antimicrobial peptides (AMPs) are a class of MAPs that kill bacteria. AMPs have multiple functions, including membrane disruption [34], inhibition of DNA synthesis [35], and cell wall synthesis in Gram-positive bacteria [36]. Therefore, it is difficult for bacteria to develop resistance to AMPs. Thus, AMPs are considered a material for solving multidrug resistance issues [37]. AMPs have an amphiphilic structure consisting of cationic amino acid residues (arginine and lysine) and hydrophobic regions; therefore, they have a positive net charge [38,39,40,41,42,43]. These cationic residues allow electrostatic interactions between the cell membrane and AMPs, and AMPs enter the hydrophobic region of the phospholipid bilayer [44]. A recent study has shown that the combination of two kinds of AMPs can significantly enhance their antimicrobial properties [45,46]. Moreover, Drab and Sugihara reported that mixtures of AMP derived from humans, such as LL-37 [47,48] and HNP1, have more effective antimicrobial activity against bacteria while minimizing host cell damage (double cooperative effects) [49]. Mixtures of two different AMPs not only enhance but also inhibit their function. Mixtures of two different AMPs may also create completely different functions.

Cell-penetrating peptides (CPPs) are another class of MAPs. CPPs have the ability to transport cargo into the living cell [61]. CPPs consist of 5-30 amino acid residues, and they have low cytotoxicity. CPPs can transport nucleotides [30], full-length proteins [62,63], small biomolecules [64], and phages [65] into living cells through covalent or non-covalent interactions. The four primary structures are cationic, amphipathic, anionic [59], and hydrophobic (Table 1). CPPs are classified using primary and secondary structures into five main classes: cationic, primary amphipathic, amphipathic α-helix, amphipathic β-sheet, and hydrophobic [66]. Cationic CPPs mainly consist of arginine and lysine: TAT (RKKRRQRRR), R8 (RRRRRRRR), and penetratin (RQIKIWFQNRRMKWKK) [50,51,52,53,54]. Primary amphipathic CPPs contain both hydrophobic and cationic domains. Some primary amphipathic CPPs are chimeric peptides, such as Pep-1 (KETWWETWWTEWSQPKKRKV) [55] and MPG (GLAFLGFLGAAGSTMGAWSQPKKKRKV) [67]. A nuclear localization signal sequence (NLS) exists in the cationic domain of these CPPs. CPPs consisting of amphipathic α-helix and amphipathic β-sheet are uniformly placed in the sequence [56,57,58]. These CPPs form secondary structures, such as VT5 (DPKGDPKGVTVTVTVTVTGKGDPKPD) [68]; these CPPs contain a highly hydrophobic region on one face and a cationic, anionic, or polar region on the other face [69]. Recently, peptide libraries have emerged as powerful tools for exploring new CPPs [70,71,72]. Hydrophobic CPPs discovered from the random peptide library, such as Pep-7, may be more efficient for interaction with the hydrophobic region of the membrane than charged CPPs derived from naturally occurring proteins and chimeric peptides [60,73].

### 2.2. Internalization Mechanism of Cell-Penetrating Peptides

**Figure 2:**
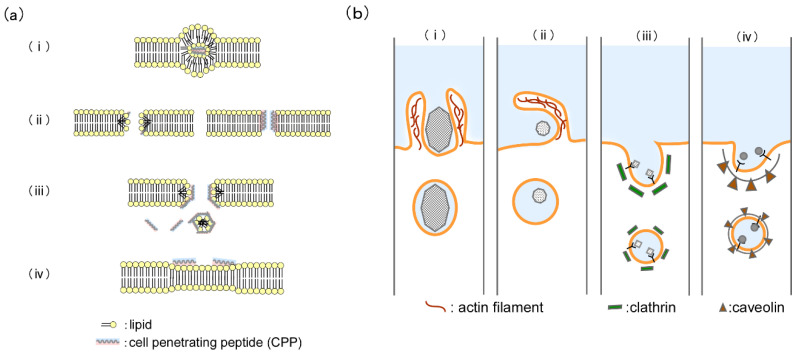
Illustration of CPP translocation mechanism. (**a**) Direct translocation pathways of CPPs: (i) inverted micelle model; (ii) pore formation model; (iii) carpet-like model; (iv) membrane-thinning model. (**b**) Endocytosis pathways of CPP: (i) phagocytosis; (ii) micropinocytosis; (iii) clathrin-mediated endocytosis; (iv) caveolae and/or lipid raft-mediated endocytosis.

The internalization mechanisms of CPPs into living cells are categorized into direct translocation and endocytosis. Direct translocation is an energy-independent pathway due to the plasma membrane potential and electrostatic interactions [74]. Four models of direct translocation are proposed, such as the inverted micelle model, pore formation model, carpet-like model, and membrane-thinning model (Figure 2a(i)–(iv)) [29,75,76,77]. In the inverted micelle model, CPPs first interact with the negatively charged molecules of the cell membrane (phospholipids, membrane proteins, or sugars). Membrane formation with an inverted structure occurs through the interaction between the hydrophobic amino acids of CPPs and the hydrophobic region of phospholipids. Therefore, direct translocation in this model can only be caused by CPPs containing cationic and hydrophobic amino acids. There are two models of pore formation formed by the CPPs: toroidal pores and barrel-stave pores. In this model, an amphiphilic α-helical peptide forms bundles on the cell membrane, and then the hydrophobic face of the peptide interacts with the phospholipid membrane. Finally, nanosized pores are formed on the cell membrane. The carpet-like model consists of three steps. First, CPPs interact with the anionic lipids of the cell membrane, and then the basic residues of CPPs are oriented on the membrane surface. Next, the hydrophobic residues of the rotating CPPs interact with the hydrophobic regions of the phospholipids. This interaction causes a minor disruption of the cell membrane. Membrane destabilization, which permits the internalization of CPPs, occurs in the inverted micelle and carpet-like models. In the membrane-thinning model, the accumulation of CPPs on the cell membrane disrupts packing in the phospholipid bilayer. The carpet-like model and membrane-thinning model translocation is caused when the CPP concentration reaches above a threshold concentration for courting the membrane surface, e.g., magaini2/lipid = 65:1 molar ratio [78].

There are four formats of endocytosis pathways, such as phagocytosis, micropinocytosis, clathrin-mediated endocytosis, and caveolae and/or lipid raft-mediated endocytosis (Figure 2b(i)–(iv)) [79]. Futaki et al. proposed that micropinocytosis is one of the major pathways involved in the internalization of arginine-rich peptides [51]. Micropinocytosis is activated by a signaling pathway that triggers actin-mediated membrane ruffling and blebbing [80]. Following the plasma membrane ruffling and blebbing, micropinosomes take particles (>0.2 µm) into the living cell. Phagocytosis is a regulatory process involved in the uptake of large particles (>0.5 µm). Clathrin-mediated endocytosis and caveolae-and/or lipid raft-mediated endocytosis are types of receptor-mediated endocytosis. During caveolae-mediated endocytosis, caveolae-coated vesicles (50–60 nm) contain small particles (~60 nm). Clathrin-mediated endocytosis takes particles (~120 nm) into the cells. These pathways depend on various CPP types, which exhibit different chemical and physical properties.

## 3. Artificial Membranes

**Figure 3:**
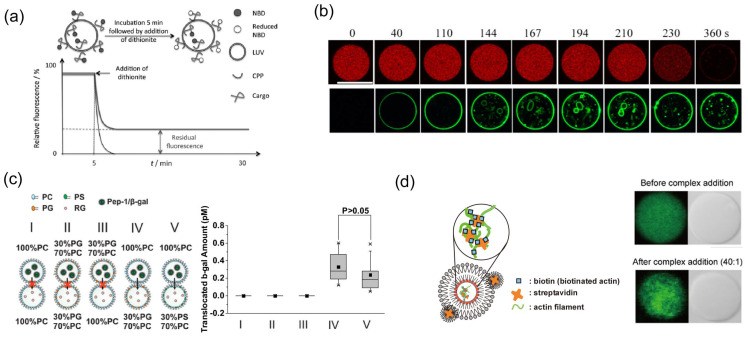
Schematic representation of CPP assay using artificial membranes. (**a**) CPP translocation assay is based on a large unilamellar vesicle (LUV) [87] *. (**b**) CPP translocation assay is based on a giant unilamellar vesicle (GUV) [88] *. (**c**) CPP-mediated protein transportation assay is based on a droplet interface bilayer (DIB) [89] *. (**d**) CPP (Pep-1, penetratin)-mediated cargo protein transportation into asymmetric GUVs [90] *. * Reproduced with permission.

The living cell membrane is composed of phospholipids, cholesterol, and membrane proteins (receptors, channels, transporters, etc.). A lipid vesicle or liposome, discovered by Bangham in the 1960s, is formed from a phospholipid bilayer [81]. Simple artificial membranes, such as lipid vesicles or liposomes, can easily be modified to change the biophysical properties of the membrane by avoiding complex reactions in biological systems. Hence, artificial membranes, such as vesicles and planar bilayers, have been used to investigate the function of membrane proteins and cellular uptake mechanisms [82,83,84,85,86]. In this section, we describe the determination of CPP characteristics, such as lipid interactions, CPP conformation, and cargo transportation using lipid vesicles and planar lipid membranes (Figure 3).

### 3.1. Observation of Conformation and Interaction Using Small Unilamellar Vesicles (SUVs) and Large Unilamellar Vesicles (LUVs)

SUVs and LUVs are mainly used to investigate the secondary structure of CPPs in the presence of phospholipids using circular dichroism spectroscopy (CD measurements) [91,92,93,94,95]. For example, TAT and the Rrg9 peptide remained disordered in the presence of SUVs containing either 1,2-di-(9*Z*-octadecenoyl)-sn-glycero-3-phospho-(1′-rac-glycerol) (DOPG), neutral phospholipids (1,2-dioleoyl-sn-glycero-3-phosphocholine; DOPC), or an 80/20 mixture of DOPC/DOPG [92]. The β-sheet structure of penetratin in the presence of SUVs containing DOPG, POPG [93], and DMPG [91] was observed. In addition, the conformational changes from a random coil structure to a β-structure depend on the concentration of PG lipids. The CD measurements revealed the electrostatic interaction between the CPP and the SUV membrane and the induction of a confocal transition of the CPP from random coil to α-helical or β-sheet forms on the SUV membranes. In other cases involving the use of LUVs, the interaction between the lipid membrane and fluorescent probe-labeled CPP was observed using fluorescence spectrophotometry (Figure 3a) [87]. The CPP translocation assay using a fluorescent probe has been performed under various conditions, such as membrane composition containing negatively charged lipids and a pH gradient using ionophores (nigericin [96], valinomycin [97,98], or membrane proteins (bacteriorhodopsin; bR)) [96,99].

### 3.2. Direct Observation of the Translocation of CPPs Using Giant Unilamellar Vesicles (GUVs)

Cell-sized lipid vesicles, for example, GUVs with a membrane composition, such as DOPC/DOPG, DOPC/chol/DOPE, and asymmetric lipid distribution, are generated using the droplet transfer method and microfluidic devices [100,101,102]. GUVs can be observed using optical microscopy. Therefore, the interaction between GUV membranes and CPPs, as well as the internalization of CPPs into GUVs, can be directly observed at the single vesicle level [103]. Viral fusion mimic GALA (sequence: WEAALAEALAEALAEHLAEALAEALEALAA)-induced fluorophore leakage inside a GUV at pH 5 was observed [59]. Recently, Md. Islam et al. proposed a method for investigating CPP internalization using a mother GUV containing small GUVs (Figure 3b). When CPPs were transported into the mother GUV, these fluorescently labeled CPPs that accumulated on the small membrane of the GUV in high concentrations were easily observed during CPP transport [104,105,106]. Furthermore, an increase in GUV curvature can be caused by external stimuli, such as osmotic pressure or micromanipulation [107]. Kazutami et al. showed that oligo-arginine, a CPP, is localized to the GUV membrane by changing the positive curvature of the GUV membrane [108]. GUVs are also used to study pore formations by MAPs. Some AMPs, such as melittin and magainin, induced pore formation in the GUV membrane composed of DOPC/DOPG [38,109]. In a CPP, the pore formation mechanism of the TAT peptide was also investigated using GUVs of DOPC, DOPG, and DOPS [110,111].

### 3.3. Observation of CPP-Mediated Cargo Transportation Using Planar Bilayer Lipid Membrane

A droplet interface bilayer (DIB) membrane, which is a planar bilayer lipid membrane, is formed between two lipid monolayers of two water-in-oil droplet interfaces based on the droplet contact method [112]. When the DIB device is connected to a patch-clamp amplifier, the ion current of the ion channel, which is reconstituted in the DIB membrane, can be recorded [113,114,115].

Gehan et al. proposed that anionic lipids (PS or PG) in the distal leaflet drive the translocation of a fluorescent probe-labeled penetratin peptide using a DIB membrane [116]. CPPs can transport oversized cargo, such as proteins and DNA, into the cytoplasm and across the cell membrane. However, the observation of a fluorescent-labeled CPP on artificial membranes has not reached a quantitative analysis of peptide-mediated protein transportation because the labeling fluorophore affects the peptide properties. Recently, Huang et al. proposed a direct method for cargo protein transport using a DIB membrane [117]. The CPP (Pep-1) and cargo (horseradish peroxidase, HRP) were mixed to create a complex formation. The CPP–cargo complexes were added to the source droplet, and only the buffer solution was added to the capture droplet. A DIB membrane was formed at the contact interface between the two types of droplets, and CPP-mediated enzyme transportation was initiated. The droplets were separated during enzyme transportation. To measure the amount of transported enzyme, the capture droplet, which was fused with another droplet containing a fluorogenic substrate, initiated the enzyme reaction. Pep-1-mediated HRP transportation was observed at a membrane potential of −50 mV. Moreover, pep-1-mediated HRP transport occurred under asymmetric membrane conditions (outer leaflets, 100% PC; inner leaflets, 90% PC and 10% PG). Xin Li et al. also obtained the high efficiency of Pep-1-mediated β-galactosidase transportation caused by the increase in PG amount in the capture droplet (Figure 3c). Therefore, the DIB membrane can be used to investigate not only the self-crossing of CPPs but also peptide-mediated protein transportation based on the direct translocation mechanisms of CPPs [89,118].

### 3.4. Application of Artificial Cell Models Using GUVs

Artificial cell models based on the self-organization of molecular building blocks (bottom-up approach) are constructed to mimic cellular behavior [119,120]. The use of CPPs in the construction of artificial cell models has also been reported. Mishra et al. showed that an FITC-labeled TAT peptide can actively induce a cytoskeletal actin response in GUVs [121]. Consequently, the actin bundles in the GUV caused their deformation. Miwa and Kamiya demonstrated the CPP-mediated direct transportation of proteins into asymmetric GUVs containing negatively charged lipids in the inner leaflet [90]. Some CPPs (Pep-1, penetratin, etc.) have a direct translocation pathway induced by a negative membrane potential (Figure 3d). This CPP-mediated protein transportation system controls the initiation of enzymatic reactions in GUVs. These studies suggest that CPPs can contribute to the development of well-defined artificial cell models integrated with membrane deformation and induction control of the protein function in GUVs.

## 4. Cargo Transportation into the Living Cell for the Control of Cellular Reactions

The delivery of biomolecules (proteins and nucleotides) to living cells plays a vital role in gene editing and cancer treatment. An encapsulation methodology for biomolecules has been developed. However, cytosolic delivery and endosomal escape remain challenging. In this section, we introduce biomolecule transportation into living cells via CPPs and a transportation system using innovative CPPs (Table 2).

### 4.1. CPP-Mediated Biomolecule Transportation into the Living Cell

When a CPP interacts significantly with biomolecules, CPP-mediated biomolecules are transported into living cells by simple mixing with the CPP and biomolecules. Protein and peptide transportation using Pep-1 involves its hydrophobic region of Pep-1 [122,124,132]. Moreover, negatively charged molecules, such as pDNA, ssDNA, and siRNA, can interact with CPPs that have positively charged amino acids through nonspecific electrostatic interactions. For example, CADY (a CPP) effectively transports siRNA into living cells because of its cationic surface. Stable CADY/siRNA complexes are obtained at a molar ratio ≥of 40/1 (CADY/siRNA) [123]. Nanomolar concentrations of CADY-mediated siRNAs can be delivered into living cells. In this non-covalent strategy, cargo, such as proteins and nucleotides, are mainly internalized into living cells via the direct transportation pathway. However, there are some restrictions to applying the non-covalent strategy, such as a high concentration of CPPs for transporting cargo and the necessity of the surface charge of the cargo.

In the covalent strategy, CPPs and cargo are conjugated via covalent bonds, such as peptide bonds (peptide linkers), disulfide bonds, sulfanyl bonds, maleimide linkers, and polyethylene glycol linkers [62,125,133,134,135]. Polyarginine and TAT have been widely studied as a covalent bond strategy. TAT(GRKKRRQRRR)-conjugated GFP at the N-terminal of GFP prepared by *Escherichia coli* expression, derived from the GST-TAT-GFP plasmid, was transferred into HeLa and CHO cells [136]. The transportation of TAT-GFP was inhibited at 4 °C. TAT-GFP was mainly transported via the caveola-mediated endocytic pathway. In general, the conjugation between CPPs and proteins may affect the biological response by reducing the affinity of the protein for the substrate in the cytoplasm. Therefore, the efficiency of cytosolic delivery (e.g., endosomal escape and direct translocation) of the cargo and the specificity of cargo delivery (e.g., targeting, and removability) using CPPs need to be developed using CPPs.

### 4.2. Increase in Endosomal Escape Efficiency in the Strategy of Non-Covalent Bonds

Non-covalent CPPs have been developed for therapeutic cargo transportation, including siRNA, Quantum Dots, antibodies, Cre protein, and Cas9 [137,138,139,140,141]. Recently, new peptides based on non-covalent complexation with the cargo have been explored, including de novo designs and peptide libraries [142]. In addition, to overcome the limitations of the endosomal release of antibodies, some non-covalent CPPs have been proposed [143,144]. For example, a lipid-sensitive endosomolytic peptide, the L17E peptide derived from M-lycotoxin (cationic membrane–lytic peptide), regulates membrane lytic activity owing to a single Glu residue on the hydrophobic face. This peptide translocates IgG from the endosome to the cytosol. This peptide does not interact with the cargo to increase the amount taken up by the cell but interacts with the endosomal membrane to efficiently release the cargo into the cell.

### 4.3. Improving Biomolecules Transportation in the Strategy of Covalent Bonds

#### 4.3.1. Enhancement of the Endosome Escape Efficiency of Cargo Molecules

**Figure 4:**
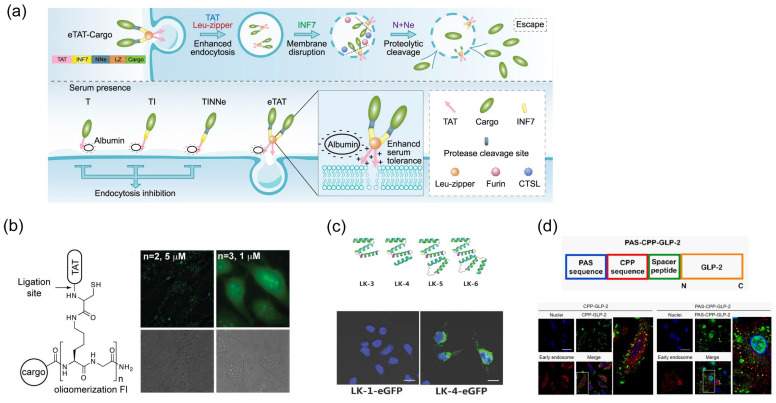
Illustration of CPP-mediated biomolecule transportation into the living cell. (**a**) eTAT system-mediated peptide fragment transportation [134] *. (**b**) One of the methods to form multivalent TAT [126] *. (**c**) Multimerization of LK peptide-mediated GFP transportation [127] *. (**d**) Penetration-accelerating site (Pas)-conjugated CPP-mediated GLP-2 transportation [128] *. * Reproduced with permission.

The low efficiency of endosomal escape for cargo delivery is a bottleneck in covalent strategies. Several strategies have been developed to enhance the endosomal escape of CPP-conjugated cargo. For example, the eTAT system comprises four modules: CPP (TAT sequence), pH-dependent membrane-active peptides (PMAPs), endosome-specific protease sites, and a leucine zipper (Figure 4a) [145]. The eTAT system delivered GFP and Protein phosphatase 1B (Ppm1b) into HEK-293T cells. To remove CPP-PMAP from the cargo protein in this eTAT system, the proteolytic cleavage site was modified between CPP-PMAP and the cargo protein. The proteolytic removal of CPP-PMAP promotes the endosomal escape of cargo proteins. Lee et al. reported that a disulfide bond between CPP and PMAP promotes endosomal escape via cytosolic cleavage [146]. These results show that the removability of CPP-PMAP plays an important role in its escape from endosomes. Moreover, the dimerization of eTAT was caused by the leucine zipper sequence in the eTAT sequence, and dimerization induces the effect of multivalent CPPs (MCPPs) with multiple copies of CPPs. MCPPs also increase endosomolytic activity. MCPPs can increase local concentrations of CPPs. High concentrations of CPPs lead to strong interactions with the cell membrane and enhance endosomal escape efficiency [147]. Several synthetic protocols for MCPPs, including the 53^tet^ (tetramerization protein)-TAT system, the branched TAT system, the squid-like (polylysine branch scaffold) TAT system, the tree-like TAT dendrimer, and multimerization of the peptide sequence, have been developed (Figure 4b,c) [126]. Jae Hoon Oh et al. showed that the multimerization of an amphipathic α-helical peptide (LKKLCKLLKKLCKLAG; leucine (L) and lysine (K)-rich α-helical (LK) peptide) accelerates the penetration rate [127]. This phenomenon was particularly observed in the tetrameric sequences. Therefore, transportation efficiency is affected by the configuration and multimerization of CPPs in the covalent strategy. CPP cyclization also increases the transportation efficiency of the cargo because cyclization increases the distance between arginine residues, thereby enhancing uptake [129,148]. Cyclic R9 delivered mCherry and the anti-GFP nanobody GBP1 to the HeLa Kyoto cells. The conjugation of the penetration-accelerating site (Pas) sequence has been previously reported [128,149]. The Pas sequence, discovered in the cleavable Cathepsin D sequence, enhances the endosomal escape efficiency of polyarginine and pAntp (N-terminally biotinylated penetratin). Pas-R8 delivered glucagon-like peptide-2 (GLP-2) into the A549 cell and the nuero-2A cell via micropinocytosis and promoted endosomal escape (Figure 4d).

#### 4.3.2. Overcoming the Lack of Specificity of Cargo Transportation

**Figure 5:**
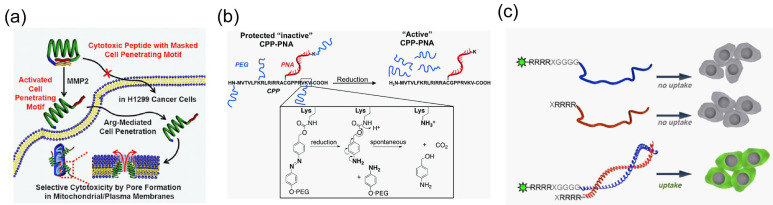
Illustration of activatable CPP (ACPP). (**a**) MMP2 activatable ACPP-mediated antimicrobial peptide transportation [156] *. (**b**) Reduction environment-sensitive ACPP that is masked by azobenzene PEG chain-mediated peptide nucleic acid (PNA) transportation [130] *. (**c**) Leucine-zipper reconstitution ACPP-mediated sf GFP transportation [131] *. * Reproduced with permission.

The low specificity of cargo delivery is another limitation of transportation via cell-penetrating peptides. Activatable CPPs (ACPPs) were first described in 2004 [150] to overcome this problem. Jiang et al. produced an ACPP via protease digestion. A CPP consists of a polyarginine domain, a cleavable linker, and polyanionic sequences as penetration inhibitors. A hairpin structure was constructed by electrostatic interactions between the polyanionic sequences and the polyarginine domain. Polyarginine is released by the cleavage of matrix metalloproteases (MMPs), which are extracellular proteases that are upregulated in cancer. Therefore, there is a specific MMP-activated polyarginine uptake by cancer cells. Many ACPP structures masked by inhibitory sequences activated by enzymes have been reported [151,152,153,154,155]. For example, Lee et al. constructed a selective cytotoxic peptide with an MMP-activatable CPP [156] that consisted of an anionic masking sequence, an MMP-cleavable linker, an antimicrobial peptide (cargo; KLA peptide), and a cationic polyarginine sequence (Figure 5a). A polyarginine sequence was conjugated to the C-terminal of the anticancer KLA peptide. The masking sequence was introduced at the other end using the MMP2 cleavable sequence as a linker. The cytotoxicity of the KLA peptide was induced by the activation of the cell-penetrating peptide. Furthermore, there are different approaches to controlling CPP activity using reduction [130], ROS-sensitive linkers [157,158], light [159,160], or the reconstitution of two short peptides [131]. Lee et al. reported a reduction in an ACPP using azobenzene PEG chains (Figure 5b). The lysine residues of the M918K peptide (MVTVLFKRLRIRRACGPPRVKV) were masked by reversible PEGylation using azobenzene. This peptide delivered peptide nucleic acids (PNAs) to HT-29-luc cells. Bode et al. investigated the reconstitution of arginine peptides derived from the leucine zipper sequence, consisting of R4(polyarginine)-VinA-sfGFP and R4-VinB (Figure 5c). This ACPP reconstitution delivers sfGFP to HeLa cells. However, unintended triggers may occur in vivo and induce off-target effects. Homing CPPs are another example of a technique to increase the selectivity of cargo transportation [161]. Several peptides that have properties of tumor cell penetration have been reported [162,163,164]. Novel homing CPPs have been isolated using mRNA display, phage display, or the random peptide library [70,165]. In addition to these methodologies, the conjugation of target ligands [166,167] and the recognition of cell-specific receptors [168] are ways to overcome the lack of specificity of cargo transportation.

## 5. Application of CPP-Mediated Control of Cellular Reactions: Functional Component Internalization

A control-released drug delivery system was proposed in 1952 with the advent of the Spansule® dissolution control (a detailed DDS review is shown in [169]). When the drug components are transferred into non-target cells, side effects will be generated. Therefore, the target delivery of the drug is essential in a therapeutic approach. The development of CPP-mediated transportation has been applied in therapy in vivo. Some of the CPP-mediated transportation of functional components regarding a therapeutic approach in vivo are shown in Table 3.

**Figure 6:**
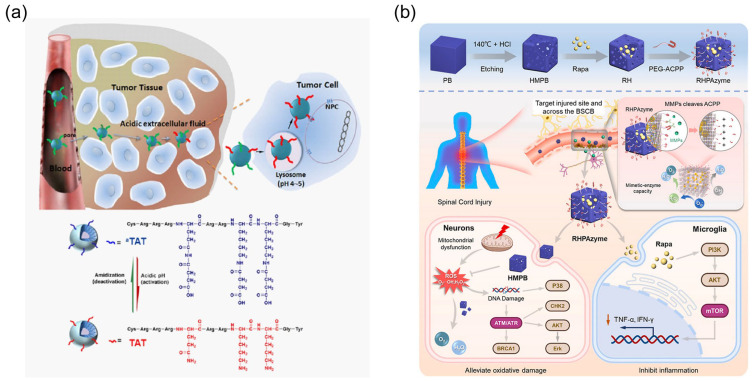
(**a**) pH-responsiveness is based on the TAT system for targeting the acidic tumor extracellular fluids [170] *. (**b**) ACPP-modified PB nanoparticles for targeting the lesion area of spinal cord injury [171] *. * Reproduced with permission.

Jin et al. developed a pH-responsive TAT system (Figure 6a) [170]. β-carboxylic amide is stable at neutral pH but hydrolyzes at acidic pH to regenerate amines. Lysine residue amines were masked by acid-labile amides to produce inactive TAT (aTAT). The anticancer drug doxorubicin (DOX) was encapsulated in aTAT micelles. When aTAT micelles were injected into mice, the tumor volume significantly decreased in the acidic tumor extracellular fluids (pH < 7). In addition, aTAT is very stable in blood and does not cause nonspecific interactions with blood components. However, a cationic CPP sometimes non-specifically interacts with negatively charged molecules in blood serum [178]. The masking of the target amino acid residues of CPP sequences affects not only tumor specificity but also the inhibition of nonspecific interactions.

Shen et al. developed an ACPP-modified nanozyme to inhibit mTOR activity (Figure 6b) [171]. The ACPP sequence is E8-PLGLAG-R9, which is cleaved by MMP-2/-9. MMPs are overexpressed in spinal cord injury (SCI) microenvironments. ACPP-modified Prussian blue (PB) nanoparticles have multi-enzyme-like activity, such as Ros scavenging activity, in the presence of rapamycin (RHPAzyme). When MMPs cleave the ACPP linker, RHPAzyme is released into the cytosol via CPPs. In an oxygen–glucose-deprived environment, RHPAzyme showed neuroprotective efficiency by scavenging ROS using PB nanoparticles and inhibiting the mTOR activity of rapamycin. In addition, ACPP-RHPAzyme targeted the injured spinal cord in mice with SCI. Thus, ACPP modification is useful for targeting typical microenvironments.

Zhang et al. developed a peptide-assisted genome editing (PAGE) system [172]. The authors proposed two hypotheses: (1) the TAT and NLS combination sequence assists cell penetration and nuclear transport, and (2) it assists chemicals, such as chloroquine and polybrene, or assists peptides (APs), such as transportan, TAT, and TAT-HA2, for endosomal escape. The PAGE system, which consists of Cas9-T2N (TAT-4 × NLS-Cas9-2 × NLS) and AP(TAT-HA2), delivers the Cas9 protein to edit the genome of primary T cells (mouse and human) and hematopoietic stem and progenitor cells (HSPCs). In addition, a single-step direct delivery of the Cas ribonucleotide protein (RNP) complex consisting of the Cas12 protein and sgRNA was achieved. After incubation for 30 min, both Cas-PAGE and Cas-RNP-PAGE showed ~100% gene-editing efficiency in primary T cells and HSPCs. Moreover, PAGE editing is less detrimental to cell viability and does not cause transcriptional changes. The PAGE system provides a platform for the ex vivo engineering of T-cell therapies using human cells.

CPP-mediated therapeutic strategies have been extended using a combination of chemical modifications or nanoparticles [173,174,175,176,177]. However, the CPP-mediated delivery of functional components must be considered for efficiency and safety in long-term applications.

## 6. Conclusions and Future Directions

This review describes methodologies for investigating CPP conformation and cargo transportation using artificial membranes (such as lipid vesicles and planar lipid membranes) and living cells. Furthermore, the improved cargo transportation systems of modified CPPs containing endosomolytic CPPs, the high endosomal escape efficiency of CPPs and ACPPs, and some therapeutic applications are introduced.

The versatility of transported cargo is one of the advantages of CPP-mediated transportation. However, the detailed mechanism of cargo transportation via CPPs is not completely understood because the mechanism of CPP-mediated cargo transportation does not simply apply to the mechanism of CPP internalization. Therefore, safety and selectivity are open to discussion for the in vivo application of CPPs. The elucidation of the CPP–cargo transportation mechanism will lead to the production of well-defined CPP systems for therapy. The use of artificial membranes, such as GUV and DIB membranes, allows the direct observation of cargo transportation. Some cargo transportation mechanisms have been proposed by observing cargo transportation using an artificial membrane. Moreover, the selectivity and stability of CPPs were increased by the modification and substitution of key functional groups or the addition of an inhibitor domain. To fill the gap in the effect between in vitro and in vivo applications, the combination of two materials, for example, ACPPs and nanoparticles, may improve the stability of the transportation for in vivo applications. Moreover, optimized CPPs that combine multi-technology, such as organic chemistry or inorganic chemistry, will be generated to provide a highly efficient and biocompatible drug delivery system.

CPP-mediated transportation is less problematic for ex vivo applications. Therefore, the ex vivo application of CPPs can be widely used for gene editing in living cells and the control of cellular functions. In addition, the application of CPP-mediated transportation will contribute not only to the functional modulation of living cells but also to the construction of artificial cells that enable the control of enzymatic reaction initiation into lipid vesicles.

## Figures and Tables

**Table 1:** Classification of typical membrane-active peptides.

	Primary	Name	Origin	Secondary	Activity (Concentration)	Target Membrane or Cell	pH, Temprature	Ref.
AMP	cationic	melitin	*Apis mellifera*	amphipathic α-helix	pore formation (≥8 nM)	DOPC or DOPG liposome	pH 7	[38]
		magainin	*Xenopus laevis*	α-helix	toroidal pore (≥10 nM)	monolayer of *E. coli* lipid extract and LUV	pH 7.4	[39]
		protegrin	*porcine neutrophils*	anti parallel β-sheet	octomer pore (25 mg/mL)	*E. coli* ML-35p cells	pH 7.4	[40]
		nisin	*Lactococcus lactis*	Loop	pore formation/inhibition of cell wall synthesis (−)	bacterial membrane	pH 2.8, 6.8 (pressure treatment)	[41]
		ndolicidin	*Bovine neutrophils*	α-helix	membrane dissolution/inhibition of DNA synthesis (10 µg/mL)	*E. coli* ML-35, *S. sureus*	pH 7.4	[42]
		Lactferricin	*human lactoferrin*	βturn/loop	direct transrocation/pore formation (≥7.5 mg/L)	*E. coli*, *S. aureus* 8532 and 8530 and so on.	pH 5–8	[43]
		LL-37	*Human*	basic/amphiphathic α-helix	pore formation/carpet model (7.5 µM)	PC/chol or PC/PS SUV and *E. coli* D21	pH 7.4, pH 8.1	[47,48]
CPP	cationic	R8	Chemic	random coil	direct transrocation/endocytosis (10 µM)	HeLa cell	pH 7 (α-MEM), 37 °C or 4 °C	[50,51]
		TAT	HIV-1-TAT protein	random coil/PpII helix	direct transrocation (500 nM)/pore formation (100 µM)	HeLa cell	pH 7 (Opt-MEM), 37 °C or 4 °C	[52,53]
		penetratin	Antennapedia homeodomain	amphipathic α-helix, β-sheet (under PG lipid)	direct translocation/endocytosis (25 µM)	E15 striatal cell	pH 7.4 (DMEM/F12), 37 °C or 4 °C	[54]
	amphipathic	Pep-1	Chimera (Trp-rich motif-SV40 NLS)	α-helix	direct translocation/water pore (0.1 µM)	HS68 fibroblasts	pH 7 (DMEM), 37 °C	[55]
		MAP	Chimeric	α-helix	Multiroute (1.8–5 µM)	Calf aortic endothelial cells (AEC)	pH 7 (DMEM), 37 °C or 0 °C	[56]
		transportan	*Galanin-mastoparan*	α-helix	endocytosis/direct translocation (5–500 nM)	Bowes’ melanoma cells	pH 7 (MEM), 37 °C or 0 °C	[57]
		pVEC	murine VE-cadherin	β-sheet	direct translocation/transporter mediated (10 µM)	AEC, HBCEC, bEND, Bowes melanoma cells	pH 7(DMEM or MEM), 37 °C or 4 °C	[58]
	anionic	GALA	Chemic (EALA repeat)	α-helix	pore formation/ membrane distavilization (2 µM)	PC LUV, POPC SUV	pH 4.5–8	[59]
	hydrophobic	Pep-7	Random Library	α-helix/homodimer	endocytosis (1 µM)	B-lymphocyte WI–L2 cells	pH 7 (RPMI 1640 medium), 37 °C	[60]

**Table 2:** Comparison of the covalent and non-covalent connection.

CPP	Cargo	Combining Strategy	Treatment Concentration (of CPP or CPP Conjugated Cargo)	Cell	Efficiency	Ref.
Pep-1	β-gal, GFP, FITC-labeled peptide	noncovalent complex	0.5 µM< [Pep-1] < 50 µM	HS-68, Cos-7 cell	>80%(protein), >90% (peptide)	[122]
CADY	short peptide, siRNA	noncovalent complex	40 or 60 µM(peptide), 1.6 µM(siRNA)	HeLa cell	unable to deliver(peptide), 97%(knockdown)	[123,124]
Cyclic R10	mcherry	disulfide bond, maleimide bond	30 µM, 50 µM	HeLa cell	ー	[125]
Branch TAT	fluorescein(FI)	carbonyl bond	1 µM, 3 µM	HeLa cell	40 %(1 µM), 80%(3 µM)	[126]
tetrameric LK-1	eGFP, PPAR	peptide bond	50 nM, 100 nM	HeLa, HEK293T cells	50%, almost 100%	[127]
PAS-CPP	Glucagon-like Peptide-2	peptide bond	6.75 µM	A549 cells	>90%	[128]
R8	TAMRA, GBP1, mcherry	disulfide bond	1 µM, 10 µM, 30 µM	HeLa Kyoto cells	5%, 90%(under free linear CPP)	[129]
activatable M918	PNA	maleimide bond	8 µM	HT-29-luc cell	60% (luciferase expression)	[130]
R4 + R4	sfGFP	peptide bond (Zipper peptide)	10 µM	HeLa cell	ー	[131]

**Table 3:** Selected examples of CPP-mediated therapeutic molecule transportation.

CPP	Composition	Cargo	Conbining Strategy	Target, Effects	Ref.
TAT	TAT-PGFK-E5	QD nanoparticles	makeimide linker	cancer (doxorubicin)	[152]
aTAT	amine masked TAT	PEG-PCL micelles	makeimide linker	tumor	[170]
R9	E8-PLGLAG-R9-Cys	PB nanoparticles	protease cleaving linker	spinal cord injury	[171]
TAT	TAT-4 × NLS-Cas9-2 × NLS, TAT-HA2	Cas9 protein	expression, mixing	genome editing	[172]
TAT	TAT (YGRKKRRQRRRC)	tandem nanomicelles	PEG linler	anti-glioma chemotherapy	[173]
R8, TAT, Penetratin	RRRRRRRR, GRKKRRQRRRPPQ, RQIKIWFQNRRMKWKK	insuline	noncovalent	Brain Delivery	[174]
TAT	GRKKRRQRRRPQPLGLAGGC	paclitaxel (PTX) prodrug nanoparticle	protease cleaving linker	Inhibition of tumor growth	[175]
R8	RRRRRRRR-hydrazone linker-ehGehGehGehG	liposome containing siRNA	hydrazone bond	gene silencing	[176]
R9	RRRRRRRR	DNA origami nanostructure	azide-alkyne cycroaddition	ros scavenger	[177]

## Data Availability

Not applicable.

## References

[1] Ye Z., Galvanetto N., Puppulin L., Pifferi S., Flechsig H., Arndt M., Triviño C.A.S., Di Palma M., Guo S., Vogel H. (2024). Structural Heterogeneity of the Ion and Lipid Channel TMEM16F. Nat. Commun..

[2] Patel V.R., Salinas A.M., Qi D., Gupta S., Sidote D.J., Goldschen-Ohm M.P. (2021). Single-Molecule Imaging with Cell-Derived Nanovesicles Reveals Early Binding Dynamics at a Cyclic Nucleotide-Gated Ion Channel. Nat. Commun..

[3] Blythe E.E., von Zastrow M. (2023). β-Arrestin-Independent Endosomal CAMP Signaling by a Polypeptide Hormone GPCR. Nat. Chem. Biol..

[4] Tang T., Tan Q., Han S., Diemar A., Löbner K., Wang H., Schüß C., Behr V., Mörl K., Wang M. (2022). Receptor-Specific Recognition of NPY Peptides Revealed by Structures of NPY Receptors. Sci. Adv..

[5] Luo P., Yu X., Wang W., Fan S., Li X., Wang J. (2015). Crystal Structure of a Phosphorylation-Coupled Vitamin C Transporter. Nat. Struct. Mol. Biol..

[6] Wu D., Chen Q., Yu Z., Huang B., Zhao J., Wang Y., Su J., Zhou F., Yan R., Li N. (2024). Transport and Inhibition Mechanisms of Human VMAT2. Nature.

[7] Mita K., Sumikama T., Iwamoto M., Matsuki Y., Shigemi K., Oiki S. (2021). Conductance Selectivity of Na^+^ across the K^+^ Channel via Na^+^ Trapped in a Tortuous Trajectory. Proc. Natl. Acad. Sci. USA.

[8] Lee H.-S., Min S., Jung Y.-E., Chae S., Heo J., Lee J.-H., Kim T., Kang H.-C., Nakanish M., Cha S.-S. (2021). Spatiotemporal Coordination of the RSF1-PLK1-Aurora B Cascade Establishes Mitotic Signaling Platforms. Nat. Commun..

[9] Sebolt-Leopold J.S., Herrera R. (2004). Targeting the Mitogen-Activated Protein Kinase Cascade to Treat Cancer. Nat. Rev. Cancer.

[10] Stipanovich A., Valjent E., Matamales M., Nishi A., Ahn J., Maroteaux M., Bertran-Gonzalez J., Brami-Cherrier K., Enslen H., Corbillé A. (2008). A Phosphatase Cascade by Which Rewarding Stimuli Control Nucleosomal Response. Nature.

[11] Valjent E., Pascoli V., Svenningsson P., Paul S., Enslen H., Corvol J.-C., Stipanovich A., Caboche J., Lombroso P.J., Nairn A.C. (2005). Regulation of a Protein Phosphatase Cascade Allows Convergent Dopamine and Glutamate Signals to Activate ERK in the Striatum. Proc. Natl. Acad. Sci. USA.

[12] Ramachandran S., Chahwan R., Nepal R.M., Frieder D., Panier S., Roa S., Zaheen A., Durocher D., Scharff M.D., Martin A. (2010). The RNF8/RNF168 Ubiquitin Ligase Cascade Facilitates Class Switch Recombination. Proc. Natl. Acad. Sci. USA.

[13] Cervia L.D., Shibue T., Borah A.A., Gaeta B., He L., Leung L., Li N., Moyer S.M., Shim B.H., Dumont N. (2023). A Ubiquitination Cascade Regulating the Integrated Stress Response and Survival in Carcinomas. Cancer Discov..

[14] Tew K.D., Manevich Y., Grek C., Xiong Y., Uys J., Townsend D.M. (2011). The Role of Glutathione S-Transferase P in Signaling Pathways and S-Glutathionylation in Cancer. Free Radic. Biol. Med..

[15] Đukić N., Strømland Ø., Elsborg J.D., Munnur D., Zhu K., Schuller M., Chatrin C., Kar P., Duma L., Suyari O. (2023). PARP14 Is a PARP with Both ADP-Ribosyl Transferase and Hydrolase Activities. Sci. Adv..

[16] Lee M.J., Yaffe M.B. (2016). Protein Regulation in Signal Transduction. Cold Spring Harb. Perspect. Biol..

[17] Liu Y., Liu S., Tomar A., Yen F.S., Unlu G., Ropek N., Weber R.A., Wang Y., Khan A., Gad M. (2023). Autoregulatory Control of Mitochondrial Glutathione Homeostasis. Science.

[18] Fakhiri J., Schneider M.A., Puschhof J., Stanifer M., Schildgen V., Holderbach S., Voss Y., El Andari J., Schildgen O., Boulant S. (2019). Novel Chimeric Gene Therapy Vectors Based on Adeno-Associated Virus and Four Different Mammalian Bocaviruses. Mol. Ther. Methods Clin. Dev..

[19] Kube S., Hersch N., Naumovska E., Gensch T., Hendriks J., Franzen A., Landvogt L., Siebrasse J.-P., Kubitscheck U., Hoffmann B. (2017). Fusogenic Liposomes as Nanocarriers for the Delivery of Intracellular Proteins. Langmuir.

[20] Hörner S., Fabritz S., Herce H.D., Avrutina O., Dietz C., Stark R.W., Cardoso M.C., Kolmar H. (2013). Cube-Octameric Silsesquioxane-Mediated Cargo Peptide Delivery into Living Cancer Cells. Org. Biomol. Chem..

[21] Cao A., Ye Z., Cai Z., Dong E., Yang X., Liu G., Deng X., Wang Y., Yang S., Wang H. (2010). A Facile Method To Encapsulate Proteins in Silica Nanoparticles: Encapsulated Green Fluorescent Protein as a Robust Fluorescence Probe. Angew. Chem. Int. Ed..

[22] Oh E.J., Park K., Kim K.S., Kim J., Yang J.-A., Kong J.-H., Lee M.Y., Hoffman A.S., Hahn S.K. (2010). Target Specific and Long-Acting Delivery of Protein, Peptide, and Nucleotide Therapeutics Using Hyaluronic Acid Derivatives. J. Control. Release.

[23] Neumann E., Schaefer-Ridder M., Wang Y., Hofschneider P.H. (1982). Gene Transfer into Mouse Lyoma Cells by Electroporation in High Electric Fields. EMBO J..

[24] Ferreira E., Potier E., Logeart-Avramoglou D., Salomskaite-Davalgiene S., Mir L.M., Petite H. (2008). Optimization of a Gene Electrotransfer Method for Mesenchymal Stem Cell Transfection. Gene Ther..

[25] REJMAN J., OBERLE V., ZUHORN I.S., HOEKSTRA D. (2004). Size-Dependent Internalization of Particles via the Pathways of Clathrin- and Caveolae-Mediated Endocytosis. Biochem. J..

[26] Hedlund H., Du Rietz H., Johansson J.M., Eriksson H.C., Zedan W., Huang L., Wallin J., Wittrup A. (2023). Single-Cell Quantification and Dose-Response of Cytosolic SiRNA Delivery. Nat. Commun..

[27] Fawell S., Seery J., Daikh Y., Moore C., Chen L.L., Pepinsky B., Barsoum J. (1994). Tat-Mediated Delivery of Heterologous Proteins into Cells. Proc. Natl. Acad. Sci. USA.

[28] Caron N.J., Torrente Y., Camirand G., Bujold M., Chapdelaine P., Leriche K., Bresolin N., Tremblay J.P. (2001). Intracellular Delivery of a Tat-EGFP Fusion Protein into Muscle Cells. Mol. Ther..

[29] Trabulo S., Cardoso A.L., Mano M., De Lima M.C.P. (2010). Cell-Penetrating Peptides—Mechanisms of Cellular Uptake and Generation of Delivery Systems. Pharmaceuticals.

[30] Khan M.M., Filipczak N., Torchilin V.P. (2021). Cell Penetrating Peptides: A Versatile Vector for Co-Delivery of Drug and Genes in Cancer. J. Control. Release.

[31] Mansur A.A.P., Carvalho S.M., Lobato Z.I.P., Leite M.D.F., Cunha A.D.S., Mansur H.S. (2018). Design and Development of Polysaccharide-Doxorubicin-Peptide Bioconjugates for Dual Synergistic Effects of Integrin-Targeted and Cell-Penetrating Peptides for Cancer Chemotherapy. Bioconjug. Chem..

[32] Kamiya K. (2020). Development of Artificial Cell Models Using Microfluidic Technology and Synthetic Biology. Micromachines.

[33] Ohnishi S., Kamiya K. (2021). Formation of Giant Lipid Vesicle Containing Dual Functions Facilitates Outer Membrane Phospholipase. ACS Synth. Biol..

[34] Chen E.H.-L., Wang C.-H., Liao Y.-T., Chan F.-Y., Kanaoka Y., Uchihashi T., Kato K., Lai L., Chang Y.-W., Ho M.-C. (2023). Visualizing the Membrane Disruption Action of Antimicrobial Peptides by Cryo-Electron Tomography. Nat. Commun..

[35] Ghosh A., Kar R.K., Jana J., Saha A., Jana B., Krishnamoorthy J., Kumar D., Ghosh S., Chatterjee S., Bhunia A. (2014). Indolicidin Targets Duplex DNA: Structural and Mechanistic Insight through a Combination of Spectroscopy and Microscopy. ChemMedChem.

[36] Wiedemann I., Breukink E., van Kraaij C., Kuipers O.P., Bierbaum G., de Kruijff B., Sahl H.-G. (2001). Specific Binding of Nisin to the Peptidoglycan Precursor Lipid II Combines Pore Formation and Inhibition of Cell Wall Biosynthesis for Potent Antibiotic Activity. J. Biol. Chem..

[37] Splith K., Neundorf I. (2011). Antimicrobial Peptides with Cell-Penetrating Peptide Properties and Vice Versa. Eur. Biophys. J..

[38] Lee M.-T., Sun T.-L., Hung W.-C., Huang H.W. (2013). Process of Inducing Pores in Membranes by Melittin. Proc. Natl. Acad. Sci. USA.

[39] Lorenzón E.N., Nobre T.M., Caseli L., Cilli E.M., da Hora G.C.A., Soares T.A., Oliveira O.N. (2018). The “Pre-Assembled State” of Magainin 2 Lysine-Linked Dimer Determines Its Enhanced Antimicrobial Activity. Colloids Surf. B Biointerfaces.

[40] Bolintineanu D., Hazrati E., Davis H.T., Lehrer R.I., Kaznessis Y.N. (2010). Antimicrobial Mechanism of Pore-Forming Protegrin Peptides: 100 Pores to Kill *E. coli*. Peptides.

[41] Modugno C., Loupiac C., Bernard A., Jossier A., Neiers F., Perrier-Cornet J.-M., Simonin H. (2018). Effect of High Pressure on the Antimicrobial Activity and Secondary Structure of the Bacteriocin Nisin. Innov. Food Sci. Emerg. Technol..

[42] Selsted M.E., Novotny M.J., Morris W.L., Tang Y.Q., Smith W., Cullor J.S. (1992). Indolicidin, a Novel Bactericidal Tridecapeptide Amide from Neutrophils. J. Biol. Chem..

[43] Jones E.M., Smart A., Bloomberg G., Burgess L., Millar M.R. (1994). Lactoferricin, a New Antimicrobial Peptide. J. Appl. Bacteriol..

[44] Li S., Wang Y., Xue Z., Jia Y., Li R., He C., Chen H. (2021). The Structure-Mechanism Relationship and Mode of Actions of Antimicrobial Peptides: A Review. Trends Food Sci. Technol..

[45] Han E., Lee H. (2015). Synergistic Effects of Magainin 2 and PGLa on Their Heterodimer Formation, Aggregation, and Insertion into the Bilayer. RSC Adv..

[46] Lee H., Lim S.I., Shin S.-H., Lim Y., Koh J.W., Yang S. (2019). Conjugation of Cell-Penetrating Peptides to Antimicrobial Peptides Enhances Antibacterial Activity. ACS Omega.

[47] Bucki R., Leszczyńska K., Namiot A., Sokołowski W. (2010). Cathelicidin LL-37: A Multitask Antimicrobial Peptide. Arch. Immunol. Ther. Exp..

[48] Oren Z., Lerman J.C., Gudmundsson G.H., Agerberth B., Shai Y. (1999). Structure and Organization of the Human Antimicrobial Peptide LL-37 in Phospholipid Membranes: Relevance to the Molecular Basis for Its Non-Cell-Selective Activity. Biochem. J..

[49] Drab E., Sugihara K. (2020). Cooperative Function of LL-37 and HNP1 Protects Mammalian Cell Membranes from Lysis. Biophys. J..

[50] Futaki S., Suzuki T., Ohashi W., Yagami T., Tanaka S., Ueda K., Sugiura Y. (2001). Arginine-Rich Peptides. J. Biol. Chem..

[51] Nakase I., Niwa M., Takeuchi T., Sonomura K., Kawabata N., Koike Y., Takehashi M., Tanaka S., Ueda K., Simpson J.C. (2004). Cellular Uptake of Arginine-Rich Peptides: Roles for Macropinocytosis and Actin Rearrangement. Mol. Ther..

[52] Vivès E., Brodin P., Lebleu B. (1997). A Truncated HIV-1 Tat Protein Basic Domain Rapidly Translocates through the Plasma Membrane and Accumulates in the Cell Nucleus. J. Biol. Chem..

[53] Rizzuti M., Nizzardo M., Zanetta C., Ramirez A., Corti S. (2015). Therapeutic Applications of the Cell-Penetrating HIV-1 Tat Peptide. Drug Discov. Today.

[54] Derossi D., Joliot A.H., Chassaing G., Prochiantz A. (1994). The Third Helix of the Antennapedia Homeodomain Translocates through Biological Membranes. J. Biol. Chem..

[55] Morris M.C., Deshayes S., Heitz F., Divita G. (2008). Cell-penetrating Peptides: From Molecular Mechanisms to Therapeutics. Biol. Cell.

[56] Oehlke J., Scheller A., Wiesner B., Krause E., Beyermann M., Klauschenz E., Melzig M., Bienert M. (1998). Cellular Uptake of an α-Helical Amphipathic Model Peptide with the Potential to Deliver Polar Compounds into the Cell Interior Non-Endocytically. Biochim. Biophys. Acta Biomembr..

[57] Pooga M., Hällbrink M., Zorko M., Langel Ü. (1998). Cell Penetration by Transportan. FASEB J..

[58] Elmquist A., Lindgren M., Bartfai T., Langel Ü. (2001). VE-Cadherin-Derived Cell-Penetrating Peptide, PVEC, with Carrier Functions. Exp. Cell Res..

[59] Li W. (2004). GALA: A Designed Synthetic PH-Responsive Amphipathic Peptide with Applications in Drug and Gene Delivery. Adv. Drug Deliv. Rev..

[60] Gao C., Mao S., Ditzel H.J., Farnaes L., Wirsching P., Lerner R.A., Janda K.D. (2002). A Cell-Penetrating Peptide from a Novel PVII–PIX Phage-Displayed Random Peptide Library. Bioorg. Med. Chem..

[61] Järver P., Langel Ü. (2006). Cell-Penetrating Peptides—A Brief Introduction. Biochim. Biophys. Acta Biomembr..

[62] Teo S.L.Y., Rennick J.J., Yuen D., Al-Wassiti H., Johnston A.P.R., Pouton C.W. (2021). Unravelling Cytosolic Delivery of Cell Penetrating Peptides with a Quantitative Endosomal Escape Assay. Nat. Commun..

[63] Niikura K., Horisawa K., Doi N. (2015). A Fusogenic Peptide from a Sea Urchin Fertilization Protein Promotes Intracellular Delivery of Biomacromolecules by Facilitating Endosomal Escape. J. Control. Release.

[64] Buyanova M., Sahni A., Yang R., Sarkar A., Salim H., Pei D. (2022). Discovery of a Cyclic Cell-Penetrating Peptide with Improved Endosomal Escape and Cytosolic Delivery Efficiency. Mol. Pharm..

[65] Zhao M., Tan X., Liu Z., Dou L., Liu D., Pan Y., Ma Y., Yu J. (2023). Engineered Phage with Cell-Penetrating Peptides for Intracellular Bacterial Infections. mSystems.

[66] Milletti F. (2012). Cell-Penetrating Peptides: Classes, Origin, and Current Landscape. Drug Discov. Today.

[67] Morris M. (1997). A New Peptide Vector for Efficient Delivery of Oligonucleotides into Mammalian Cells. Nucleic Acids Res..

[68] Oehlke J., Krause E., Wiesner B., Beyermann M., Bienert M. (1997). Extensive Cellular Uptake into Endothelial Cells of an Amphipathic Β-sheet Forming Peptide. FEBS Lett..

[69] Gong Z., Ikonomova S.P., Karlsson A.J. (2018). Secondary Structure of Cell-penetrating Peptides during Interaction with Fungal Cells. Protein Sci..

[70] Kondo E., Saito K., Tashiro Y., Kamide K., Uno S., Furuya T., Mashita M., Nakajima K., Tsumuraya T., Kobayashi N. (2012). Tumour Lineage-Homing Cell-Penetrating Peptides as Anticancer Molecular Delivery Systems. Nat. Commun..

[71] Numata K., Horii Y., Oikawa K., Miyagi Y., Demura T., Ohtani M. (2018). Library Screening of Cell-Penetrating Peptide for BY-2 Cells, Leaves of Arabidopsis, Tobacco, Tomato, Poplar, and Rice Callus. Sci. Rep..

[72] Oikawa K., Islam M.M., Horii Y., Yoshizumi T., Numata K. (2018). Screening of a Cell-Penetrating Peptide Library in Escherichia Coli: Relationship between Cell Penetration Efficiency and Cytotoxicity. ACS Omega.

[73] Marks J.R., Placone J., Hristova K., Wimley W.C. (2011). Spontaneous Membrane-Translocating Peptides by Orthogonal High-Throughput Screening. J. Am. Chem. Soc..

[74] Di Pisa M., Chassaing G., Swiecicki J.M. (2015). Translocation Mechanism(s) of Cell-Penetrating Peptides: Biophysical Studies Using Artificial Membrane Bilayers. Biochemistry.

[75] Madani F., Lindberg S., Langel Ü., Futaki S., Gräslund A. (2011). Mechanisms of Cellular Uptake of Cell-Penetrating Peptides. J. Biophys..

[76] Heitz F., Morris M.C., Divita G. (2009). Twenty Years of Cell-penetrating Peptides: From Molecular Mechanisms to Therapeutics. Br. J. Pharmacol..

[77] Ruseska I., Zimmer A. (2020). Internalization Mechanisms of Cell-Penetrating Peptides. Beilstein J. Nanotechnol..

[78] Ludtke S., He K., Huang H. (1995). Membrane Thinning Caused by Magainin 2. Biochemistry.

[79] Schmid S.L., Conner S.D. (2003). Regulated Portals of Entry into the Cell. Nature.

[80] Mercer J., Helenius A. (2009). Virus Entry by Macropinocytosis. Nat. Cell Biol..

[81] Bangham A.D., Horne R.W. (1964). Negative Staining of Phospholipids and Their Structural Modification by Surface-Active Agents as Observed in the Electron Microscope. J. Mol. Biol..

[82] Tosaka T., Kamiya K. (2023). Function Investigations and Applications of Membrane Proteins on Artificial Lipid Membranes. Int. J. Mol. Sci..

[83] Kamiya K. (2022). Formation and Function of OmpG or OmpA-Incorporated Liposomes Using an in Vitro Translation System. Sci. Rep..

[84] Kawano R., Tsuji Y., Sato K., Osaki T., Kamiya K., Hirano M., Ide T., Miki N., Takeuchi S. (2013). Automated Parallel Recordings of Topologically Identified Single Ion Channels. Sci. Rep..

[85] Kamiya K., Osaki T., Nakao K., Kawano R., Fujii S., Misawa N., Hayakawa M., Takeuchi S. (2018). Electrophysiological Measurement of Ion Channels on Plasma/Organelle Membranes Using an on-Chip Lipid Bilayer System. Sci. Rep..

[86] Guha A., McGuire M.L., Leriche G., Yang J., Mayer M. (2021). A Single-Liposome Assay That Enables Temperature-Dependent Measurement of Proton Permeability of Extremophile-Inspired Lipid Membranes. Biochim. Biophys. Acta Biomembr..

[87] Swiecicki J., Bartsch A., Tailhades J., Di Pisa M., Heller B., Chassaing G., Mansuy C., Burlina F., Lavielle S. (2014). The Efficacies of Cell-Penetrating Peptides in Accumulating in Large Unilamellar Vesicles Depend on Their Ability To Form Inverted Micelles. ChemBioChem.

[88] Islam M.Z., Ariyama H., Alam J.M., Yamazaki M. (2014). Entry of Cell-Penetrating Peptide Transportan 10 into a Single Vesicle by Translocating Across Lipid Membrane and Its Induced Pores. Biochemistry.

[89] Li X., Huang J., Holden M.A., Chen M. (2017). Peptide-Mediated Membrane Transport of Macromolecular Cargo Driven by Membrane Asymmetry. Anal. Chem..

[90] Miwa A., Kamiya K. (2022). Control of Enzyme Reaction Initiation inside Giant Unilamellar Vesicles by the Cell-Penetrating Peptide-Mediated Translocation of Cargo Proteins. ACS Synth. Biol..

[91] Magzoub M., Kilk K., Eriksson L.E.G., Langel Ü., Gräslund A. (2001). Interaction and Structure Induction of Cell-Penetrating Peptides in the Presence of Phospholipid Vesicles. Biochim. Biophys. Acta Biomembr..

[92] Eiríksdóttir E., Konate K., Langel Ü., Divita G., Deshayes S. (2010). Secondary Structure of Cell-Penetrating Peptides Controls Membrane Interaction and Insertion. Biochim. Biophys. Acta Biomembr..

[93] Magzoub M., Eriksson L.E.G., Gräslund A. (2002). Conformational States of the Cell-Penetrating Peptide Penetratin When Interacting with Phospholipid Vesicles: Effects of Surface Charge and Peptide Concentration. Biochim. Biophys. Acta Biomembr..

[94] Christiaens B., Grooten J., Reusens M., Joliot A., Goethals M., Vandekerckhove J., Prochiantz A., Rosseneu M. (2004). Membrane Interaction and Cellular Internalization of Penetratin Peptides. Eur. J. Biochem..

[95] Gonçalves F., Castro T.G., Nogueira E., Pires R., Silva C., Ribeiro A., Cavaco-Paulo A. (2018). OBP Fused with Cell-Penetrating Peptides Promotes Liposomal Transduction. Colloids Surfaces B Biointerfaces.

[96] Magzoub M., Pramanik A., Gräslund A. (2005). Modeling the Endosomal Escape of Cell-Penetrating Peptides: Transmembrane PH Gradient Driven Translocation across Phospholipid Bilayers. Biochemistry.

[97] Terrone D., Sang S.L.W., Roudaia L., Silvius J.R. (2003). Penetratin and Related Cell-Penetrating Cationic Peptides Can Translocate Across Lipid Bilayers in the Presence of a Transbilayer Potential. Biochemistry.

[98] Henriques S.T., Costa J., Castanho M.A.R.B. (2005). Translocation of β-Galactosidase Mediated by the Cell-Penetrating Peptide Pep-1 into Lipid Vesicles and Human HeLa Cells Is Driven by Membrane Electrostatic Potential. Biochemistry.

[99] Björklund J., Biverståhl H., Gräslund A., Mäler L., Brzezinski P. (2006). Real-Time Transmembrane Translocation of Penetratin Driven by Light-Generated Proton Pumping. Biophys. J..

[100] Pautot S., Frisken B.J., Weitz D.A. (2003). Engineering Asymmetric Vesicles. Proc. Natl. Acad. Sci. USA.

[101] Kamiya K., Osaki T., Takeuchi S. (2019). Formation of Vesicles-in-a-Vesicle with Asymmetric Lipid Components Using a Pulsed-Jet Flow Method. RSC Adv..

[102] Lu L., Schertzer J.W., Chiarot P.R. (2015). Continuous Microfluidic Fabrication of Synthetic Asymmetric Vesicles. Lab Chip.

[103] Bárány-Wallje E., Keller S., Serowy S., Geibel S., Pohl P., Bienert M., Dathe M. (2005). A Critical Reassessment of Penetratin Translocation Across Lipid Membranes. Biophys. J..

[104] Walrant A., Matheron L., Cribier S., Chaignepain S., Jobin M., Sagan S., Alves I.D. (2013). Direct Translocation of Cell-Penetrating Peptides in Liposomes: A Combined Mass Spectrometry Quantification and Fluorescence Detection Study. Anal. Biochem..

[105] Islam M.Z., Sharmin S., Moniruzzaman M., Yamazaki M. (2018). Elementary Processes for the Entry of Cell-Penetrating Peptides into Lipid Bilayer Vesicles and Bacterial Cells. Appl. Microbiol. Biotechnol..

[106] Shuma M.L., Moghal M.M.R., Yamazaki M. (2020). Detection of the Entry of Nonlabeled Transportan 10 into Single Vesicles. Biochemistry.

[107] Islam M.Z., Sharmin S., Levadnyy V., Alam Shibly S.U., Yamazaki M. (2017). Effects of Mechanical Properties of Lipid Bilayers on the Entry of Cell-Penetrating Peptides into Single Vesicles. Langmuir.

[108] Sakamoto K., Morishita T., Aburai K., Ito D., Imura T., Sakai K., Abe M., Nakase I., Futaki S., Sakai H. (2021). Direct Entry of Cell-Penetrating Peptide Can Be Controlled by Maneuvering the Membrane Curvature. Sci. Rep..

[109] Billah M.M., Or Rashid M.M., Ahmed M., Yamazaki M. (2023). Antimicrobial Peptide Magainin 2-Induced Rupture of Single Giant Unilamellar Vesicles Comprising *E. coli* Polar Lipids. Biochim. Biophys. Acta Biomembr..

[110] Ciobanasu C., Siebrasse J.P., Kubitscheck U. (2010). Cell-Penetrating HIV1 TAT Peptides Can Generate Pores in Model Membranes. Biophys. J..

[111] Sun S., Xia Y., Liu J., Dou Y., Yang K., Yuan B., Kang Z. (2022). Real-Time Monitoring the Interfacial Dynamic Processes at Model Cell Membranes: Taking Cell Penetrating Peptide TAT as an Example. J. Colloid Interface Sci..

[112] Funakoshi K., Suzuki H., Takeuchi S. (2006). Lipid Bilayer Formation by Contacting Monolayers in a Microfluidic Device for Membrane Protein Analysis. Anal. Chem..

[113] Gotanda M., Kamiya K., Osaki T., Fujii S., Misawa N., Miki N., Takeuchi S. (2018). Sequential Generation of Asymmetric Lipid Vesicles Using a Pulsed-Jetting Method in Rotational Wells. Sens. Actuators B Chem..

[114] Tsuji Y., Kawano R., Osaki T., Kamiya K., Miki N., Takeuchi S. (2013). Droplet Split-and-Contact Method for High-Throughput Transmembrane Electrical Recording. Anal. Chem..

[115] Kamiya K., Arisaka C., Suzuki M. (2021). Investigation of Fusion between Nanosized Lipid Vesicles and a Lipid Monolayer Toward Formation of Giant Lipid Vesicles with Various Kinds of Biomolecules. Micromachines.

[116] Gehan P., Kulifaj S., Soule P., Bodin J.B., Amoura M., Walrant A., Sagan S., Thiam A.R., Ngo K., Vivier V. (2020). Penetratin Translocation Mechanism through Asymmetric Droplet Interface Bilayers. Biochim. Biophys. Acta Biomembr..

[117] Huang J., Lein M., Gunderson C., Holden M. (2011). a Direct Quantitation of Peptide-Mediated Protein Transport across a Droplet–Interface Bilayer. J. Am. Chem. Soc..

[118] Lein M., DeRonde B.M., Sgolastra F., Tew G.N., Holden M.A. (2015). Protein Transport across Membranes: Comparison between Lysine and Guanidinium-Rich Carriers. Biochim. Biophys. Acta Biomembr..

[119] Kamiya K., Osaki T., Takeuchi S. (2021). Formation of Nano-Sized Lipid Vesicles with Asymmetric Lipid Components Using a Pulsed-Jet Flow Method. Sens. Actuators B Chem..

[120] Kamiya K., Takeuchi S. (2017). Giant Liposome Formation toward the Synthesis of Well-Defined Artificial Cells. J. Mater. Chem. B.

[121] Mishra A., Lai G.H., Schmidt N.W., Sun V.Z., Rodriguez A.R., Tong R., Tang L., Cheng J., Deming T.J., Kamei D.T. (2011). Translocation of HIV TAT Peptide and Analogues Induced by Multiplexed Membrane and Cytoskeletal Interactions. Proc. Natl. Acad. Sci. USA.

[122] Morris M., Depollier J., Heitz F., Divita G. (2006). A Peptide Carrier for the Delivery of Biologically Active Proteins into Mammalian CellsApplication to the Delivery of Antibodies and Therapeutic Proteins. Cell Biology.

[123] Crombez L., Aldrian-Herrada G., Konate K., Nguyen Q.N., McMaster G.K., Brasseur R., Heitz F., Divita G. (2009). A New Potent Secondary Amphipathic Cell-Penetrating Peptide for SiRNA Delivery into Mammalian Cells. Mol. Ther..

[124] Kurzawa L., Pellerano M., Morris M.C. (2010). PEP and CADY-Mediated Delivery of Fluorescent Peptides and Proteins into Living Cells. Biochim. Biophys. Acta Biomembr..

[125] Schneider A.F.L., Wallabregue A.L.D., Franz L., Hackenberger C.P.R. (2019). Targeted Subcellular Protein Delivery Using Cleavable Cyclic Cell-Penetrating Peptides. Bioconjug. Chem..

[126] Angeles-Boza A.M., Erazo-Oliveras A., Lee Y.-J., Pellois J.-P. (2010). Generation of Endosomolytic Reagents by Branching of Cell-Penetrating Peptides: Tools for the Delivery of Bioactive Compounds to Live Cells in Cis or Trans. Bioconjug. Chem..

[127] Oh J.H., Chong S., Nam S., Hyun S., Choi S., Gye H., Jang S., Jang J., Hwang S.W., Yu J. (2018). Multimeric Amphipathic A-Helical Sequences for Rapid and Efficient Intracellular Protein Transport at Nanomolar Concentrations. Adv. Sci..

[128] Akita T., Kimura R., Akaguma S., Nagai M., Nakao Y., Tsugane M., Suzuki H., Oka J., Yamashita C. (2021). Usefulness of Cell-Penetrating Peptides and Penetration Accelerating Sequence for Nose-to-Brain Delivery of Glucagon-like Peptide-2. J. Control. Release.

[129] Schneider A.F.L., Kithil M., Cardoso M.C., Lehmann M., Hackenberger C.P.R. (2021). Cellular Uptake of Large Biomolecules Enabled by Cell-Surface-Reactive Cell-Penetrating Peptide Additives. Nat. Chem..

[130] Lee S.H., Moroz E., Castagner B., Leroux J.-C. (2014). Activatable Cell Penetrating Peptide–Peptide Nucleic Acid Conjugate via Reduction of Azobenzene PEG Chains. J. Am. Chem. Soc..

[131] Bode S.A., Kruis I.C., Adams H.P.J.H.M., Boelens W.C., Pruijn G.J.M., van Hest J.C.M., Löwik D.W.P.M. (2017). Coiled-Coil-Mediated Activation of Oligoarginine Cell-Penetrating Peptides. ChemBioChem.

[132] Morris M.C., Depollier J., Mery J., Heitz F., Divita G. (2001). A Peptide Carrier for the Delivery of Biologically Active Proteins into Mammalian Cells. Nat. Biotechnol..

[133] Imesch P., Scheiner D., Szabo E., Fink D., Fedier A. (2013). Conjugates of Cytochrome c and Antennapedia Peptide Activate Apoptosis and Inhibit Proliferation of HeLa Cancer Cells. Exp. Ther. Med..

[134] Zamaleeva A.I., Collot M., Bahembera E., Tisseyre C., Rostaing P., Yakovlev A.V., Oheim M., de Waard M., Mallet J.-M., Feltz A. (2014). Cell-Penetrating Nanobiosensors for Pointillistic Intracellular Ca^2+^ -Transient Detection. Nano Lett..

[135] Qian Z., Xu X., Amacher J.F., Madden D.R., Cormet-Boyaka E., Pei D. (2015). Intracellular Delivery of Peptidyl Ligands by Reversible Cyclization: Discovery of a PDZ Domain Inhibitor That Rescues CFTR Activity. Angew. Chem. Int. Ed..

[136] Ferrari A., Pellegrini V., Arcangeli C., Fittipaldi A., Giacca M., Beltram F. (2003). Caveolae-Mediated Internalization of Extracellular HIV-1 Tat Fusion Proteins Visualized in Real Time. Mol. Ther..

[137] Liu Y., Song Z., Zheng N., Nagasaka K., Yin L., Cheng J. (2018). Systemic SiRNA Delivery to Tumors by Cell-Penetrating α-Helical Polypeptide-Based Metastable Nanoparticles. Nanoscale.

[138] van Asbeck A.H., Beyerle A., McNeill H., Bovee-Geurts P.H.M., Lindberg S., Verdurmen W.P.R., Hällbrink M., Langel Ü., Heidenreich O., Brock R. (2013). Molecular Parameters of SiRNA–Cell Penetrating Peptide Nanocomplexes for Efficient Cellular Delivery. ACS Nano.

[139] Kawaguchi Y., Kawamura Y., Hirose H., Kiyokawa M., Hirate M., Hirata T., Higuchi Y., Futaki S. (2024). E3MPH16: An Efficient Endosomolytic Peptide for Intracellular Protein Delivery. J. Control. Release.

[140] Liu B.R., Huang Y.W., Winiarz J.G., Chiang H.J., Lee H.J. (2011). Intracellular Delivery of Quantum Dots Mediated by a Histidine- and Arginine-Rich HR9 Cell-Penetrating Peptide through the Direct Membrane Translocation Mechanism. Biomaterials.

[141] Del’Guidice T., Lepetit-Stoffaes J.-P., Bordeleau L.-J., Roberge J., Théberge V., Lauvaux C., Barbeau X., Trottier J., Dave V., Roy D.-C. (2018). Membrane Permeabilizing Amphiphilic Peptide Delivers Recombinant Transcription Factor and CRISPR-Cas9/Cpf1 Ribonucleoproteins in Hard-to-Modify Cells. PLoS ONE.

[142] de Oliveira E.C.L., Santana K., Josino L., Lima e Lima A.H., de Souza de Sales Júnior C. (2021). Predicting Cell-Penetrating Peptides Using Machine Learning Algorithms and Navigating in Their Chemical Space. Sci. Rep..

[143] Akishiba M., Takeuchi T., Kawaguchi Y., Sakamoto K., Yu H.H., Nakase I., Takatani-Nakase T., Madani F., Gräslund A., Futaki S. (2017). Cytosolic Antibody Delivery by Lipid-Sensitive Endosomolytic Peptide. Nat. Chem..

[144] Allen J.K., Brock D.J., Kondow-McConaghy H.M., Pellois J.-P. (2018). Efficient Delivery of Macromolecules into Human Cells by Improving the Endosomal Escape Activity of Cell-Penetrating Peptides: Lessons Learned from DfTAT and Its Analogs. Biomolecules.

[145] Yu S., Yang H., Li T., Pan H., Ren S., Luo G., Jiang J., Yu L., Chen B., Zhang Y. (2021). Efficient Intracellular Delivery of Proteins by a Multifunctional Chimaeric Peptide in Vitro and in Vivo. Nat. Commun..

[146] Lee Y.-J., Johnson G., Peltier G.C., Pellois J.-P. (2011). A HA2-Fusion Tag Limits the Endosomal Release of Its Protein Cargo despite Causing Endosomal Lysis. Biochim. Biophys. Acta Gen. Subj..

[147] Erazo-Oliveras A., Muthukrishnan N., Baker R., Wang T.-Y., Pellois J.-P. (2012). Improving the Endosomal Escape of Cell-Penetrating Peptides and Their Cargos: Strategies and Challenges. Pharmaceuticals.

[148] Nischan N., Herce H.D., Natale F., Bohlke N., Budisa N., Cardoso M.C., Hackenberger C.P.R. (2015). Covalent Attachment of Cyclic TAT Peptides to GFP Results in Protein Delivery into Live Cells with Immediate Bioavailability. Angew. Chem. Int. Ed..

[149] Takayama K., Nakase I., Michiue H., Takeuchi T., Tomizawa K., Matsui H., Futaki S. (2009). Enhanced Intracellular Delivery Using Arginine-Rich Peptides by the Addition of Penetration Accelerating Sequences (Pas). J. Control. Release.

[150] Jiang T., Olson E.S., Nguyen Q.T., Roy M., Jennings P.A., Tsien R.Y. (2004). Tumor Imaging by Means of Proteolytic Activation of Cell-Penetrating Peptides. Proc. Natl. Acad. Sci. USA.

[151] Olson E.S., Jiang T., Aguilera T.A., Nguyen Q.T., Ellies L.G., Scadeng M., Tsien R.Y. (2010). Activatable Cell Penetrating Peptides Linked to Nanoparticles as Dual Probes for in Vivo Fluorescence and MR Imaging of Proteases. Proc. Natl. Acad. Sci. USA.

[152] Li J., Liu F., Shao Q., Min Y., Costa M., Yeow E.K.L., Xing B. (2014). Enzyme-Responsive Cell-Penetrating Peptide Conjugated Mesoporous Silica Quantum Dot Nanocarriers for Controlled Release of Nucleus-Targeted Drug Molecules and Real-Time Intracellular Fluorescence Imaging of Tumor Cells. Adv. Healthc. Mater..

[153] Zhao N., Bardine C., Lourenço A.L., Wang Y., Huang Y., Cleary S.J., Wilson D.M., Oh D.Y., Fong L., Looney M.R. (2021). In Vivo Measurement of Granzyme Proteolysis from Activated Immune Cells with PET. ACS Cent. Sci..

[154] Hingorani D.V., Camargo M.F., Quraishi M.A., Adams S.R., Advani S.J. (2021). Tumor Activated Cell Penetrating Peptides to Selectively Deliver Immune Modulatory Drugs. Pharmaceutics.

[155] van Duijnhoven S.M.J., Robillard M.S., Nicolay K., Grüll H. (2011). Tumor Targeting of MMP-2/9 Activatable Cell-Penetrating Imaging Probes Is Caused by Tumor-Independent Activation. J. Nucl. Med..

[156] Lee J., Oh E.-T., Lee H.-J., Lee E., Kim H.G., Park H.J., Kim C. (2022). Tuning of Peptide Cytotoxicity with Cell Penetrating Motif Activatable by Matrix Metalloproteinase-2. ACS Omega.

[157] Weinstain R., Savariar E.N., Felsen C.N., Tsien R.Y. (2014). In Vivo Targeting of Hydrogen Peroxide by Activatable Cell-Penetrating Peptides. J. Am. Chem. Soc..

[158] Perez-Lopez A.M., Valero E., Bradley M. (2017). Synthesis and Optimization of a Reactive Oxygen Species Responsive Cellular Delivery System. New J. Chem..

[159] Hansen M.B., Van Gaal E., Minten I., Storm G., Van Hest J.C.M., Löwik D.W.P.M. (2012). Constrained and UV-Activatable Cell-Penetrating Peptides for Intracellular Delivery of Liposomes. J. Control. Release.

[160] Lin Y., Mazo M.M., Skaalure S.C., Thomas M.R., Simon R. (2018). Activatable Cell-Biomaterial Interfacing with Photo-Caged Peptides. Chem. Sci..

[161] Liu Z., Wang F., Chen X. (2011). Integrin Targeted Delivery of Chemotherapeutics. Theranostics.

[162] Myrberg H., Zhang L., Mäe M., Langel Ü. (2008). Design of a Tumor-Homing Cell-Penetrating Peptide. Bioconjug. Chem..

[163] Allred C.A., Gormley C., Venugopal I., Li S., McGuire M.J., Brown K.C. (2023). Tumor-Specific Intracellular Delivery: Peptide-Guided Transport of a Catalytic Toxin. Commun. Biol..

[164] Zahid M., Phillips B.E., Albers S.M., Giannoukakis N., Watkins S.C., Robbins P.D. (2010). Identification of a Cardiac Specific Protein Transduction Domain by In Vivo Biopanning Using a M13 Phage Peptide Display Library in Mice. PLoS ONE.

[165] Higa M., Katagiri C., Shimizu-Okabe C., Tsumuraya T., Sunagawa M., Nakamura M., Ishiuchi S., Takayama C., Kondo E., Matsushita M. (2015). Identification of a Novel Cell-Penetrating Peptide Targeting Human Glioblastoma Cell Lines as a Cancer-Homing Transporter. Biochem. Biophys. Res. Commun..

[166] Youn P., Chen Y., Furgeson D.Y. (2014). A Myristoylated Cell-Penetrating Peptide Bearing a Transferrin Receptor-Targeting Sequence for Neuro-Targeted SiRNA Delivery. Mol. Pharm..

[167] Laroui N., Cubedo N., Rossel M., Bettache N. (2020). Improvement of Cell Penetrating Peptide for Efficient SiRNA Targeting of Tumor Xenografts in Zebrafish Embryos. Adv. Ther..

[168] Tan M., Lan K.-H., Yao J., Lu C.-H., Sun M., Neal C.L., Lu J., Yu D. (2006). Selective Inhibition of ErbB2-Overexpressing Breast Cancer In Vivo by a Novel TAT-Based ErbB2-Targeting Signal Transducers and Activators of Transcription 3–Blocking Peptide. Cancer Res..

[169] Park H., Otte A., Park K. (2022). Evolution of Drug Delivery Systems: From 1950 to 2020 and Beyond. J. Control. Release.

[170] Jin E., Zhang B., Sun X., Zhou Z., Ma X., Sun Q., Tang J., Shen Y., Van Kirk E., Murdoch W.J. (2013). Acid-Active Cell-Penetrating Peptides for in Vivo Tumor-Targeted Drug Delivery. J. Am. Chem. Soc..

[171] Shen K., Li X., Huang G., Yuan Z., Xie B., Chen T., He L. (2023). High Rapamycin-Loaded Hollow Mesoporous Prussian Blue Nanozyme Targets Lesion Area of Spinal Cord Injury to Recover Locomotor Function. Biomaterials.

[172] Zhang Z., Baxter A.E., Ren D., Qin K., Chen Z., Collins S.M., Huang H., Komar C.A., Bailer P.F., Parker J.B. (2024). Efficient Engineering of Human and Mouse Primary Cells Using Peptide-Assisted Genome Editing. Nat. Biotechnol..

[173] Khafagy E.S., Morishita M. (2012). Oral Biodrug Delivery Using Cell-Penetrating Peptide. Adv. Drug Deliv. Rev..

[174] Zhu Y., Jiang Y., Meng F., Deng C., Cheng R., Zhang J., Feijen J., Zhong Z. (2018). Highly Efficacious and Specific Anti-Glioma Chemotherapy by Tandem Nanomicelles Co-Functionalized with Brain Tumor-Targeting and Cell-Penetrating Peptides. J. Control. Release.

[175] Yin H., Lu H., Xiong Y., Ye L., Teng C., Cao X., Li S., Sun S., Liu W., Lv W. (2021). Tumor-Associated Neutrophil Extracellular Traps Regulating Nanocarrier-Enhanced Inhibition of Malignant Tumor Growth and Distant Metastasis. ACS Appl. Mater. Interfaces.

[176] Xiang B., Jia X.-L., Qi J.-L., Yang L.-P., Sun W.-H., Yan X., Yang S.-K., Cao D.-Y., Du Q., Qi X.-R. (2017). Enhancing SiRNA-Based Cancer Therapy Using a New PH-Responsive Activatable Cell-Penetrating Peptide-Modified Liposomal System. Int. J. Nanomed..

[177] Wang H., Jiao Y., Ma S., Li Z., Gong J., Jiang Q., Shang Y., Li H., Li J., Li N. (2024). Nebulized Inhalation of Peptide-Modified DNA Origami To Alleviate Acute Lung Injury. Nano Lett..

[178] Kosuge M., Takeuchi T., Nakase I., Jones A.T., Futaki S. (2008). Cellular Internalization and Distribution of Arginine-Rich Peptides as a Function of Extracellular Peptide Concentration, Serum, and Plasma Membrane Associated Proteoglycans. Bioconjug. Chem..

